# Identification of the miRNA-mRNA regulatory network associated with radiosensitivity in esophageal cancer based on integrative analysis of the TCGA and GEO data

**DOI:** 10.1186/s12920-022-01392-9

**Published:** 2022-12-01

**Authors:** Hongmin Chen, Xiaoxiao Shi, Li Ren, Hongyu Zhuo, Li Zeng, Qing Qin, Yuming Wan, Wangmu Sangdan, Lin Zhou

**Affiliations:** 1 Cancer Center, West China Hospital of Sichuan University, No. 37 Guoxue Lane, Wuhou District, Chengdu, 610041 People’s Republic of China; 2 Department of Medical Oncology, Chengdu Shang Jin Nan Fu Hospital (West China Hospital, S.C.U.), Chengdu, 611730 People’s Republic of China; 3 Department of Oncology, People’s Hospital of Tibet Autonomous Region, Lhasa, 850000 People’s Republic of China; 4 Department of Thoracic Oncology, State Key Laboratory of Biotherapy, Sichuan University, No. 1, Keyuan 4th Road, Gaopeng Avenue, Chengdu, 610041 People’s Republic of China

**Keywords:** Esophageal cancer, Radiosensitivity, miRNA-mRNA regulatory network, miR-132-3p, CAND1, ZDHHC23, miR-576-5p, AHR

## Abstract

**Background:**

The current study set out to identify the miRNA-mRNA regulatory networks that influence the radiosensitivity in esophageal cancer based on the The Cancer Genome Atlas (TCGA) and the Gene Expression Omnibus (GEO) databases.

**Methods:**

Firstly, esophageal cancer-related miRNA-seq and mRNA-seq data were retrieved from the TCGA database, and the mRNA dataset of esophageal cancer radiotherapy was downloaded from the GEO database to analyze the differential expressed miRNAs (DEmiRNAs) and mRNAs (DEmRNAs) in radiosensitive and radioresistant samples, followed by the construction of the miRNA-mRNA regulatory network and Gene Ontology and KEGG enrichment analysis. Additionally, a prognostic risk model was constructed, and its accuracy was evaluated by means of receiver operating characteristic analysis.

**Results:**

A total of 125 DEmiRNAs and 42 DEmRNAs were closely related to the radiosensitivity in patients with esophageal cancer. Based on 47 miRNA-mRNA interactions, including 21 miRNAs and 21 mRNAs, the miRNA-mRNA regulatory network was constructed. The prognostic risk model based on 2 miRNAs (miR-132-3p and miR-576-5p) and 4 mRNAs (CAND1, ZDHHC23, AHR, and MTMR4) could accurately predict the prognosis of esophageal cancer patients. Finally, it was verified that miR-132-3p/CAND1/ZDHHC23 and miR-576-5p/AHR could affect the radiosensitivity in esophageal cancer.

**Conclusion:**

Our study demonstrated that miR-132-3p/CAND1/ZDHHC23 and miR-576-5p/AHR were critical molecular pathways related to the radiosensitivity of esophageal cancer.

**Supplementary Information:**

The online version contains supplementary material available at 10.1186/s12920-022-01392-9.

## Background

Esophageal cancer, as one of the leading malignancies in China, is accompanied by a high incidence of morbidity (varies among geographic areas) and mortality rates, with the overall 5-year survival rate ranging from 15 to 25% worldwide [1]. The onset of esophageal cancer is characterized by numerous unspecific symptoms, including loss of appetite, weight loss, dysphagia, and nausea, which makes timely diagnosis a clinical challenge [2]. Currently, radiotherapy represents the mainstay regimen for the treatment esophageal cancer [3]. Despite the tremendous advancements made in regard to radiotherapy, the side-effects of radiotherapy and radiation resistance still remain the chief obstacle in clinical practice [4]. Accordingly, it is imperative to explore novel molecular biomarkers for increasing the radiosensitivity of esophageal cancer, and further improve the prognosis and treatment of patients with esophageal cancer.

As highly recognized, a new strategy provided by system biology with a specific focus on gene expression profiles can aid the discovery of novel molecular targets and prognostic factors [5]. With the development of systems biology, its application in the field of cancer has resulted in cancer systems biology, and possesses the potential for better understanding of the underlying mechanisms of cancer occurrence and progression, and on the diagnosis and treatment of cancer [6, 7]. Moreover, cancer systems biology holds great responsibility in cancer treatment primarily through the integration of bioinformatics-related content and joint diagnostic methods for the screening, classification, assessment of cancer progression and treatment response, and individualized and systemic treatment of cancer [8].

MicroRNAs (miRNAs) are capable of modulating gene expression at a post-translational level by directly-binding to target mRNAs, and also implicated in the development of diverse human cancers *via* mediation of cell biology, including esophageal cancer [9, 10]. What’s more, miRNAs are known to function as biomarkers to influence radiosensitive/radioresistance in esophageal cancer [11]. Numerous studies have attempted to explore the roles of miRNAs in enhancing the radiosensitivity of esophageal cancer cells by virtue of regulating their target mRNAs [12, 13]. For instance, a previous prognostic risk model incorporated three miRNA and target genes and highlighted their association with the prognosis of patients and critical immunological effects in the progression and metastasis of esophageal cancer [9].

Cancer is one of the leading causes of death worldwide and is a global burden despite the development of diagnosis, treatment modalities or potential prevention methods [14]. Diverse treatment modalities have been developed, but even the most commonly used chemotherapeutic agents are accompanied by multiple serious adverse effects, including neutropenia, sensory neuropathy and diarrhea, cardiotoxicity, nephrotoxicity, gastrointestinal toxicity, cardiovascular toxicity, pulmonary toxicity and hematologic toxicity, poor bioavailability, poor safety and limited effectiveness [15]. Many efforts have been made to limit adverse effects during cancer treatment, such as the identification of common molecular origins of various diseases through developments of genomics, proteomics and informatics technologies, as well as the analytical tools [16].

Herein, our research group aimed to establish a holistic landscape of gene interaction networks in radiosensitive and radioresistant samples through gene expression profiles retrieved from microarray datasets. Accordingly, we carried out a series of comprehensive analyses, including univariate Cox analysis, the least absolute shrinkage and selection operator (LASSO) regression analysis, multivariate Cox analysis, and receiver operating characteristic (ROC) analysis, to focus on differential expressed miRNAs (DEmiRNAs) and mRNAs (DEmRNAs) downloaded from the TCGA and GEO database. Subsequent findings in our study uncovered the miRNA-mRNA regulatory network associated with the radiosensitivity in esophageal cancer, which holds the potential of predicting the prognosis of patients with esophageal cancer.

## Materials and methods

### Data sources

**Fig. 1:**
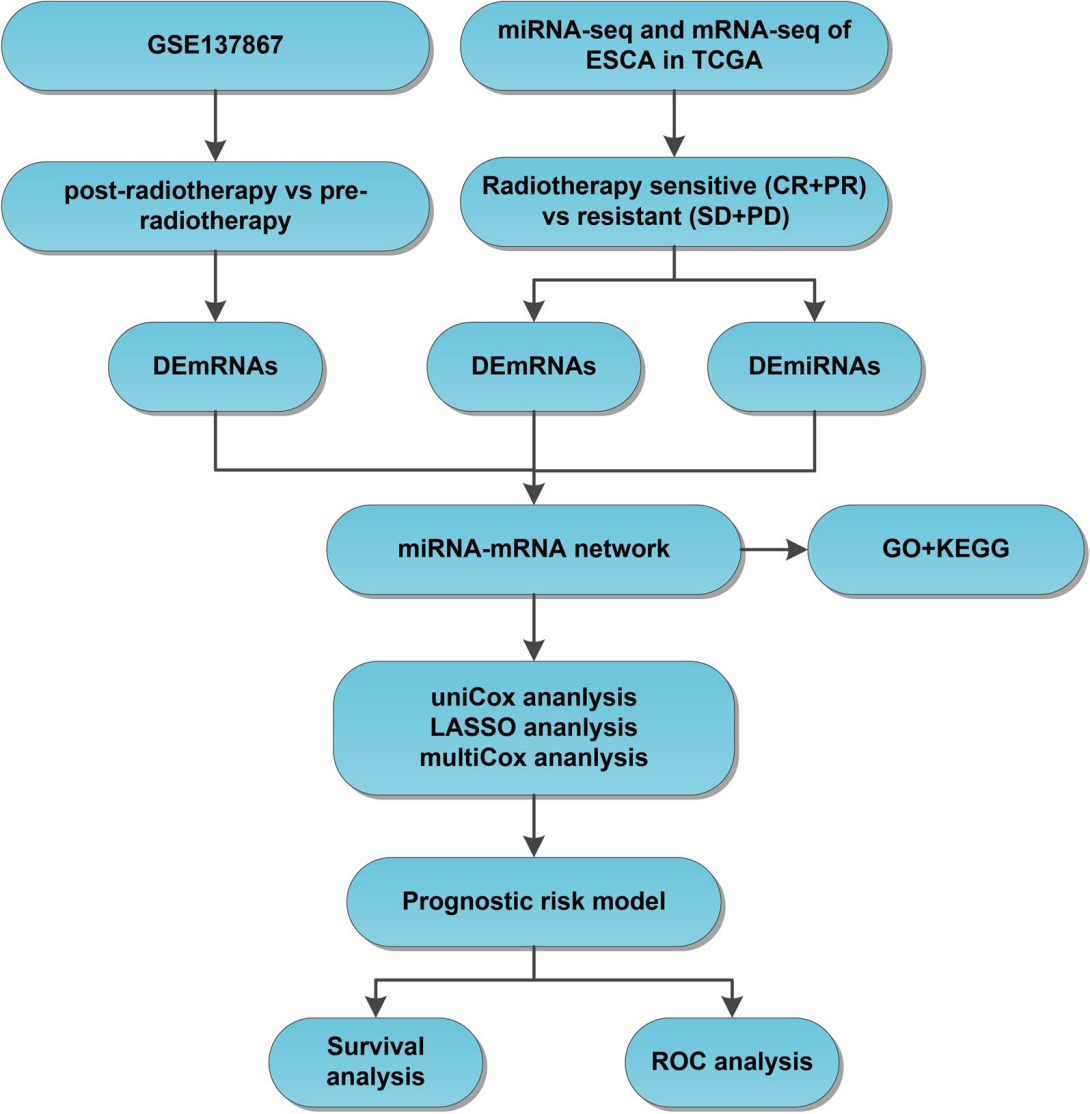
Flow chart of the analysis in the current study. The miRNA-seq and mRNA-seq data of esophageal cancer were obtained from the TCGA database, and the expression differences of miRNAs and mRNAs in radiosensitive samples and radioresistant samples were analyzed, and the radiotherapy-related mRNA dataset of esophageal cancer was obtained from the GEO database. Then, DEmRNAs before and after radiotherapy were obtained. After that, the miRNA-mRNA regulatory networks were constructed through the online databases, and GO and KEGG enrichment analyses were utilized to predict the biological functions involved in mRNAs. A prognostic risk model was constructed using univariate Cox regression analysis, LASSO regression analysis and multivariate Cox regression analysis. Finally, a ROC curve analysis was performed to evaluate the predictive accuracy of candidate factors

The flow chart of the analysis in the current study is illustrated in Fig. 1. The esophageal cancer-related miRNA-seq, mRNA-seq, and clinical data in the TCGA program were retrieved from the UCSC Xena database (http://xena.ucsc.edu). The format of downloaded expression profile data is Fragments per Kilobase Million. In the expression profiles of selected patients with esophageal cancer receiving radiotherapy, complete remission/response (CR) and partial remission/response (PR) were classified in the radiosensitive group, while stable disease (SD) and progressive disease (PD) were classified in the radioresistant group. The RNA-seq data from 31 esophageal cancer samples (comprising of 25 radiosensitive samples and 6 radioresistant samples) and miRNA-seq data from 36 esophageal cancer samples (comprising of 30 radiosensitive samples and 6 radioresistant samples) met the requirements. In addition, mRNA dataset GSE137867 (comprising of 4 esophageal cancer samples with pre-radiotherapy and 4 esophageal cancer samples with post-radiotherapy) was further downloaded with the keyword “esophageal cancer radiotherapy” from the GEO database (http://www.ncbi.nlm.nih.goc/geo/) as a validation dataset.

### Differential expression analysis

The “limma” package of R software (https://bioconductor.org/packages/limma/) was adopted to screen the DEmiRNAs and DEmRNAs, with *p* < 0.05 serving as the screening threshold. The normalization process was performed using the normalizeBetweenArrays() function of the limma package. Next, the DEmRNAs in the GSE137867 dataset with the consistent expression in the TCGA were further analyzed.

### Prediction of the miRNA-mRNA regulatory network

The miRNA-mRNA interactions were predicted with the help of the TargetScan (http://www.targetscan.org/), ENCORI (https://starbase.sysu.edu.cn/index.php), and RNAInter (http://www.rna-society.org/rnainter/) datasets, respectively. The target mRNAs consistently identified by these three databases were selected as the candidate target genes, and further intersected with DEmRNAs to screen the potential target mRNAs of miRNAs. The miRNA-mRNA regulatory network was visualized using the Cytoscape software (http://cytoscape.org/, version 3.8.2).

### Gene ontology (GO) and kyoto encyclopedia of genes and genomes (KEGG) enrichment analysis

The enrichment analysis of GO and KEGG function [17–19] was conducted using the “ClusterProfiler” package of R software (http://www.bioconductor.org/packages/release/bioc/html/clusterProfiler.html). A value of *p* < 0.05 was regarded statistically significant.

### Construction of the prognostic risk model

The expression profiles of DEmRNAs and DEmiRNAs in the miRNA-mRNA regulatory network were retrieved from patients with esophageal cancer in the TCGA program. Subsequently, a univariate COX analysis was performed using the “survival” package of R language (http://bioconductor.org/packages/survival/) to identify the prognosis-related DEmRNAs and DEmiRNAs. Next, a LASSO analysis was conducted based on the “glmnet” package (https://CRAN.R-project.org/package=glmnet). Thereafter, a multivariate COX analysis was carried out to establish a prognostic risk model, and the risk score of each patient under the regression model was calculated as follows: risk score = the regression coefficient of each gene * the sum of its expression level. Patients were further classified into high- and low-risk groups based on their median risk score, and a Kaplan-Meier survival analysis was performed to compare the overall survival (OS) differences between high-and low-risk groups with *p* < 0.05 serving as the cutoff value. Receiver operating characteristic (ROC) analysis was conducted using the “timeROC” (https://cran.r-project.org/web/packages/timeROC/index.html) and ROC curves were drawn to assess the accuracy of the prognostic risk model.

### Statistical analysis

All statistical analyses were performed using the R software (version 4.1.1; R Foundation for Statistical Computing, Vienna, Austria). Comparison between groups was analyzed by means of unpaired *t*-test. A value of *p* < 0.05 was regarded statistically significant.

## Results

### Identification of DEmiRNAs and DEmRNAs related to the radiosensitivity in patients with esophageal cancer

**Fig. 2:**
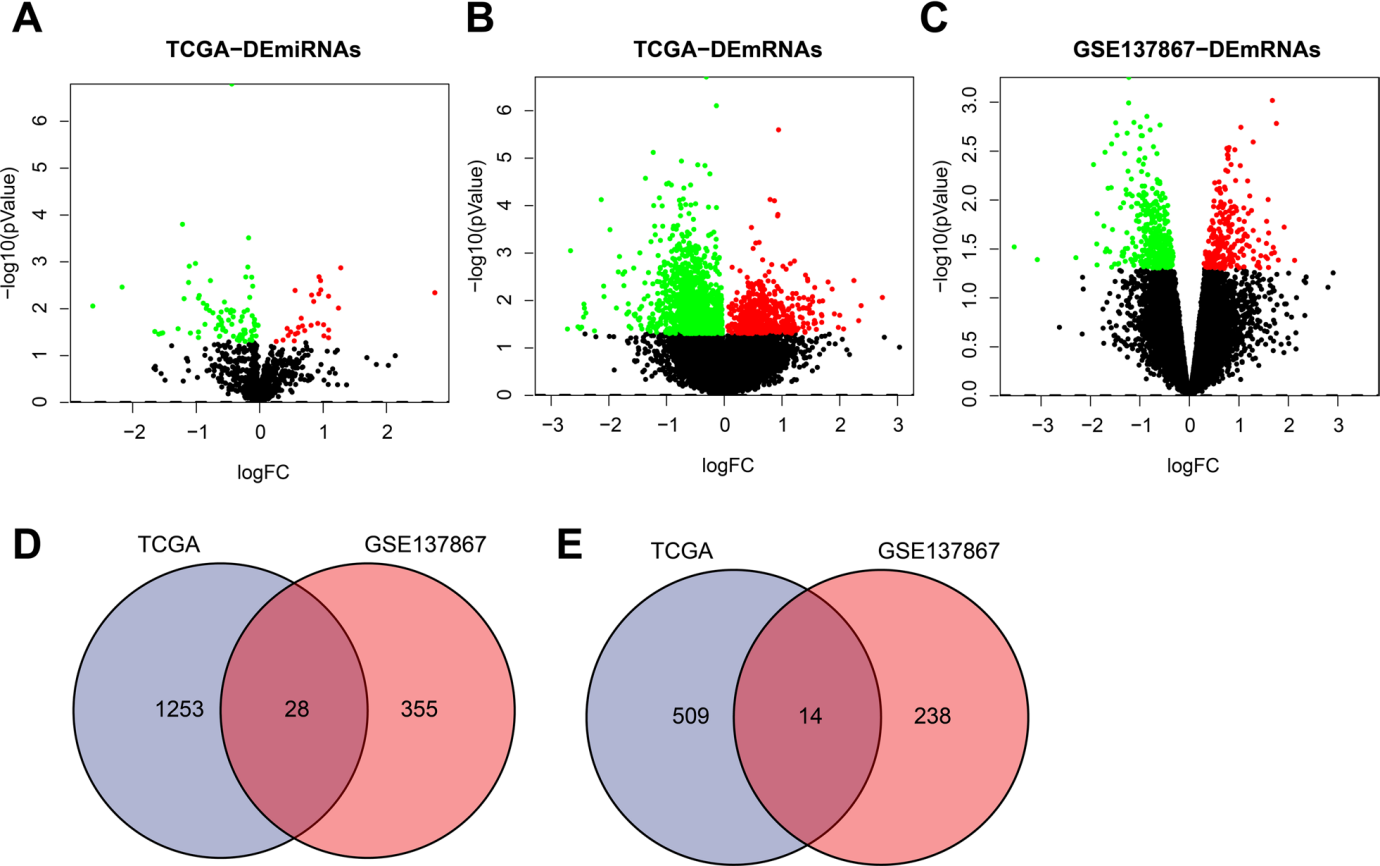
Identification of the radiosensitivity-related DEmiRNAs and DEmRNAs in patients with esophageal cancer. **A** Volcano map of DEmiRNAs between radiosensitive and radioresistant groups in TCGA, Red dots indicate upregulated miRNAs, green dots indicate downregulated miRNAs, and black dots indicate miRNAs without differential expression. **B** Volcano map of DEmRNAs between radiosensitive and radioresistant groups in TCGA, Red dots indicate upregulated mRNAs, green dots indicate downregulated mRNAs, and black dots indicate mRNAs without differential expression. **C** Volcano map of DEmRNAs from four patients with esophageal cancer before and after radiotherapy in GSE137867 dataset, Red dots indicate upregulated mRNAs, green dots indicate downregulated mRNAs, and black dots indicate mRNAs without differential expression. **D** Venn diagram of intersection of downregulated DEmRNAs between TCGA and GSE137867 dataset. **E** Venn diagram of intersection of upregulated DEmRNAs between TCGA and GSE137867 dataset

Differential expression patterns of miRNAs and mRNAs between radiosensitive and radioresistant groups were compared based on the TCGA database to explore the radiosensitivity-related miRNAs and mRNAs. Subsequent results highlighted a total of 125 DEmiRNAs (including 29 up-regulated and 96 down-regulated miRNAs) and 1782 DEmRNAs (including 523 up-regulated mRNAs and 1259 down-regulated mRNAs) (Fig. 2A, B).

Additionally, we retrieved a total of 18 related datasets by searching the keyword “esophageal cancer radiotherapy” in the GEO database. After the filter condition was set to “Expression profiling by array”, a total of 9 related datasets were obtained. The relevant information is illustrated in Additional file 1: Table S1. After that, the expression profiles of four patients with esophageal cancer before and after radiotherapy in the GSE137867 dataset were compared. Thereafter, we uncovered 118 differentially expressed genes (DEGs), of which 50 genes were up-regulated and 67 genes were down-regulated (Fig. 2C). The overlapping DEmRNAs between the TCGA and GSE137867 dataset were integrated and 42 DEGs were obtained, comprising of 28 down-regulated and 14 up-regulated genes (Fig. 2D, E).

Overall, these results indicated that the aforementioned 125 DEmiRNAs and 42 DEmRNAs may be closely associated with the radiosensitivity in patients with esophageal cancer.

### Construction of the miRNA-mRNA regulatory network related to the radiosensitivity in esophageal cancer

**Fig. 3:**
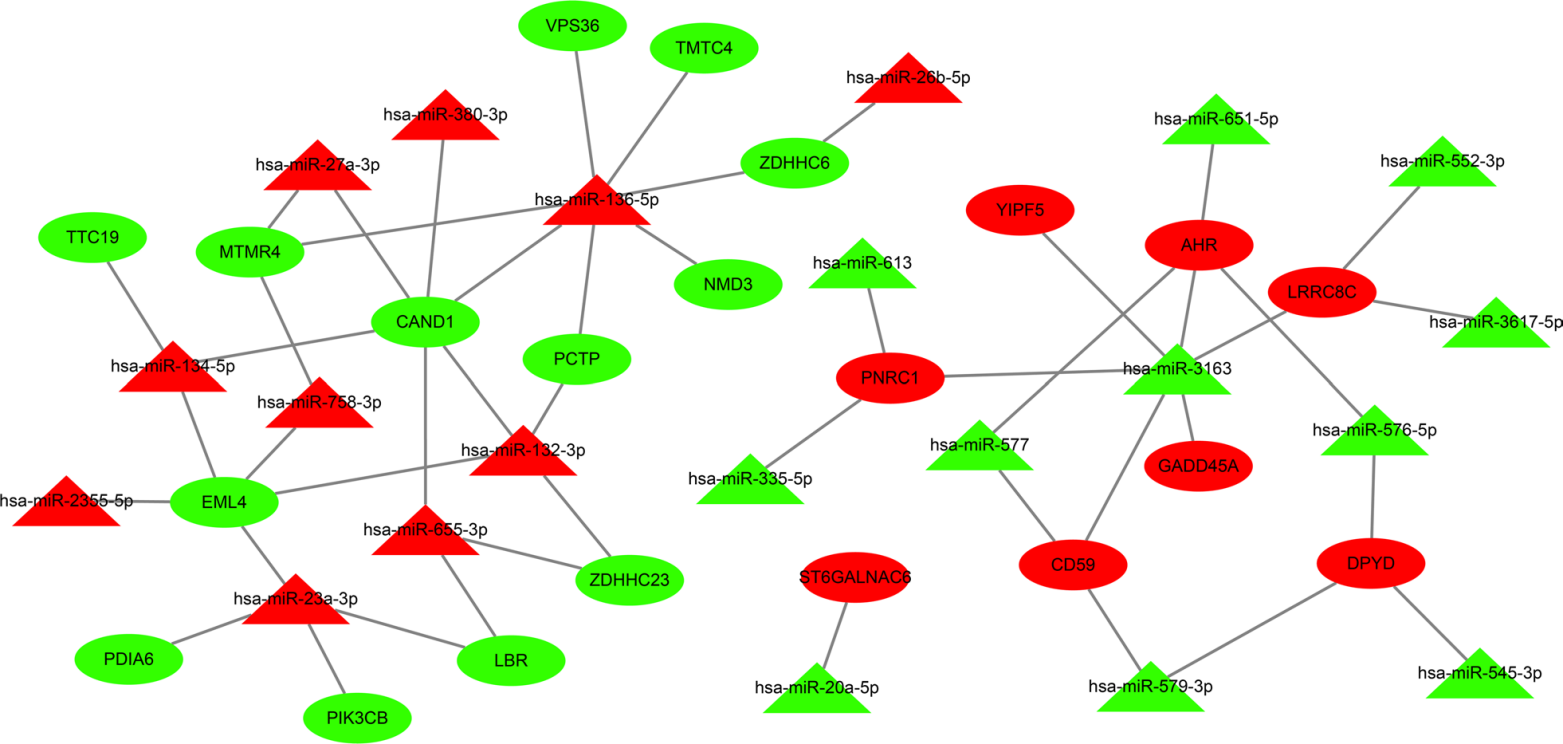
Construction of the miRNA-mRNA regulatory network affecting the radiosensitivity in esophageal cancer using Cytoscape software. The triangles refer to miRNAs, and the circles refer to mRNA. Red indicates upregulated genes in radiosensitive group, and green indicates downregulated genes in radiosensitive group

Thereafter, a miRNA-mRNA regulatory network was further constructed to investigate the mechanisms that could influence radiosensitivity in esophageal cancer. Accumulating studies have established with the negative-correlation between miRNA and target genes [20]. Herein, the relationship between 125 DEmiRNAs and 42 DEmRNAs was assessed by the TargetScan, ENCORI, and RNAInter databases, which identified 47 miRNA-mRNA interactions, including 21 miRNAs and 21 mRNAs. Subsequently, the miRNA-mRNA regulatory network was constructed using the Cytoscape software (Fig. 3).

### Functional enrichment analysis of DEmRNAs in the miRNA-mRNA regulatory network associated with radiosensitivity in esophageal cancer

**Fig. 4:**
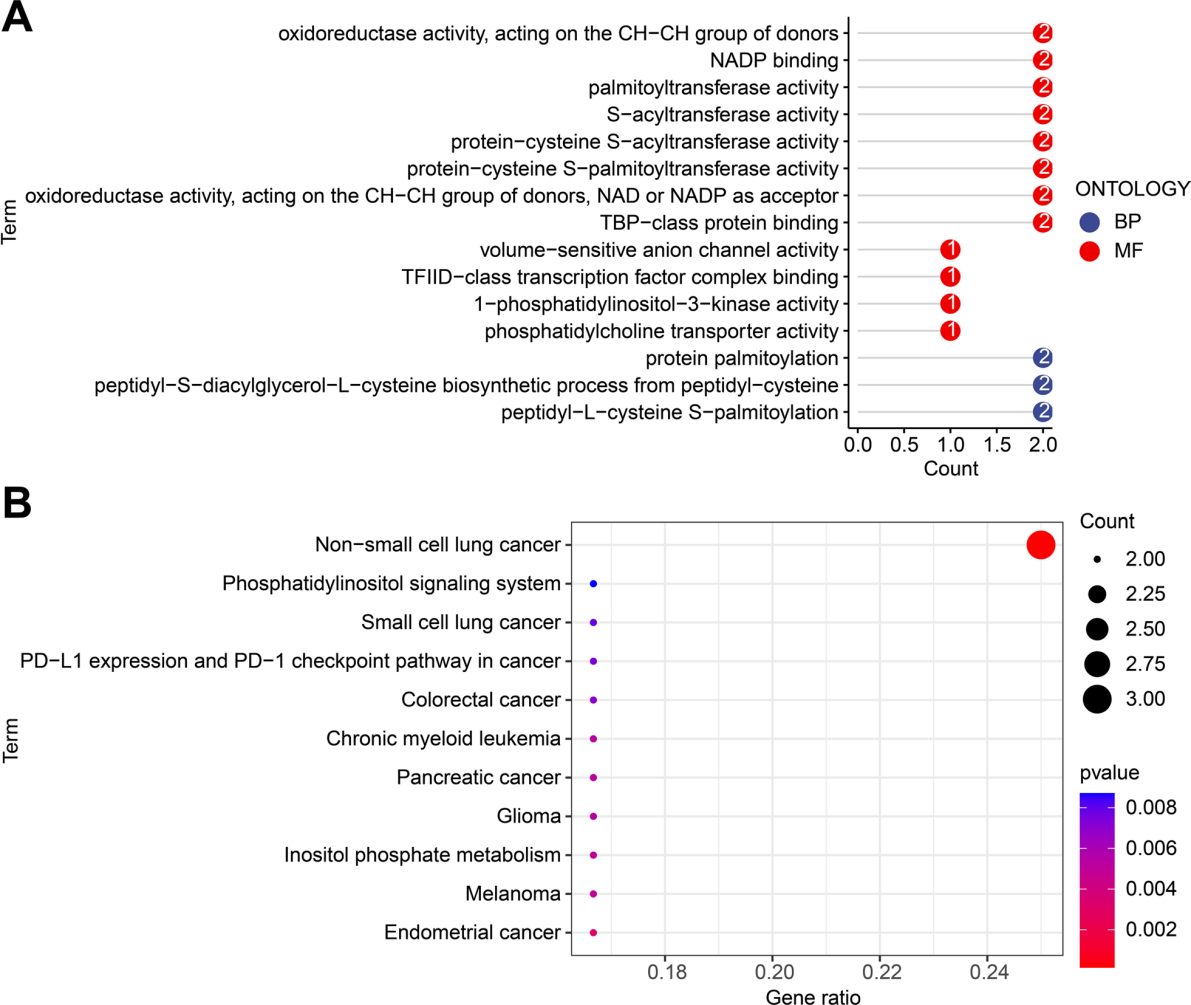
DEmRNAs in the miRNA-mRNA regulatory network involved in the radiosensitivity in esophageal cancer based on GO and KEGG enrichment analysis. **A** GO functional enrichment analysis of DEmRNAs. **B** KEGG enrichment analysis of DEmRNAs

Additionally, GO and KEGG enrichment analyses were carried out on 21 DEmRNAs to better elucidate the biological function of DEmRNAs in the miRNA-mRNA regulatory network associated with radiosensitivity in esophageal cancer. Subsequent results demonstrated that mRNAs were primarily enriched in molecular function (MF) and biological processes (BP), and further related to protein palmitoylation and oxidoreductase activity (Fig. 4A). Results of KEGG analysis also revealed that mRNAs were enriched in various cancers (Fig. 4B).

Together, these results suggested that DEmRNAs in the miRNA-mRNA regulatory network may participate in the occurrence and development of esophageal cancer.

### **The prognostic risk model based on 2 miRNAs and 4 mRNAs could accurately predict the prognosis of patients with esophageal cancer**

**Fig. 5:**
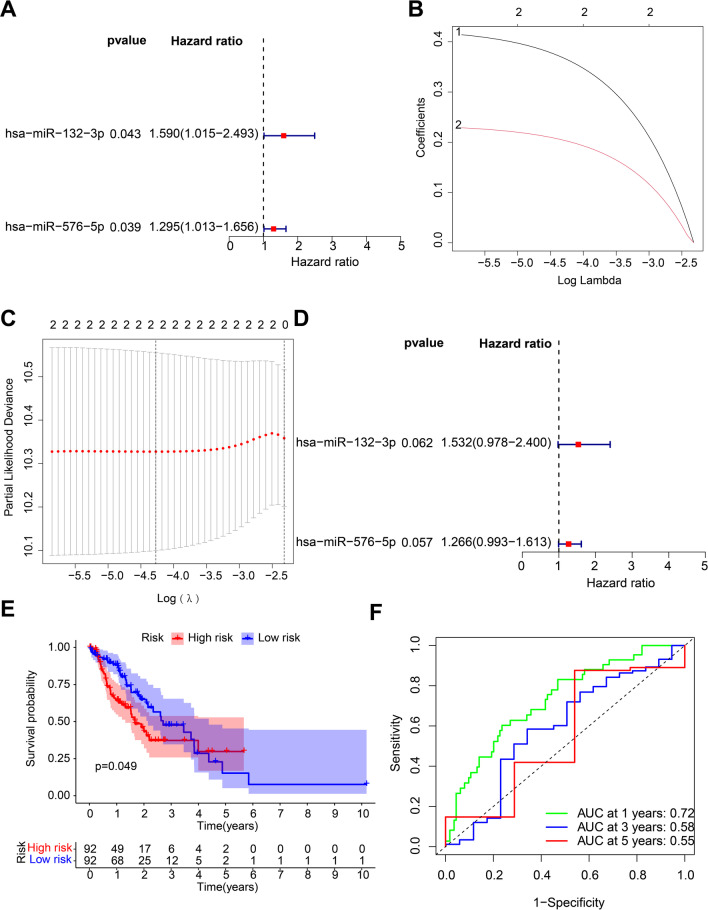
Evaluation of efficacy of the radiosensitivity-related miRNAs in the prognosis of patients with esophageal cancer. **A** Univariate Cox regression analysis for the prognosis-related miRNAs in esophageal cancer patients. The left part indicates the miRNA name, and the middle part indicates the *p* value. The Hazard ratio represents the risk rate. Risk rate greater than 1 represents a high risk of this gene, and risk rate less than 1 represents a low risk. The right part indicates the risk rate distribution. **B** LASSO coefficient distribution of 2 miRNAs in esophageal cancer. **C** Selection of the optimal parameters (lambda) in the LASSO analysis of esophageal cancer. **D** Multivariate Cox regression analysis for the prognosis-related miRNAs in esophageal cancer patients. **E** A Kaplan-Meier survival curve analysis of patients with high- and low-risk. **F** ROC analysis of the prognostic risk model. The lower right corner indicates the AUC value

**Fig. 6:**
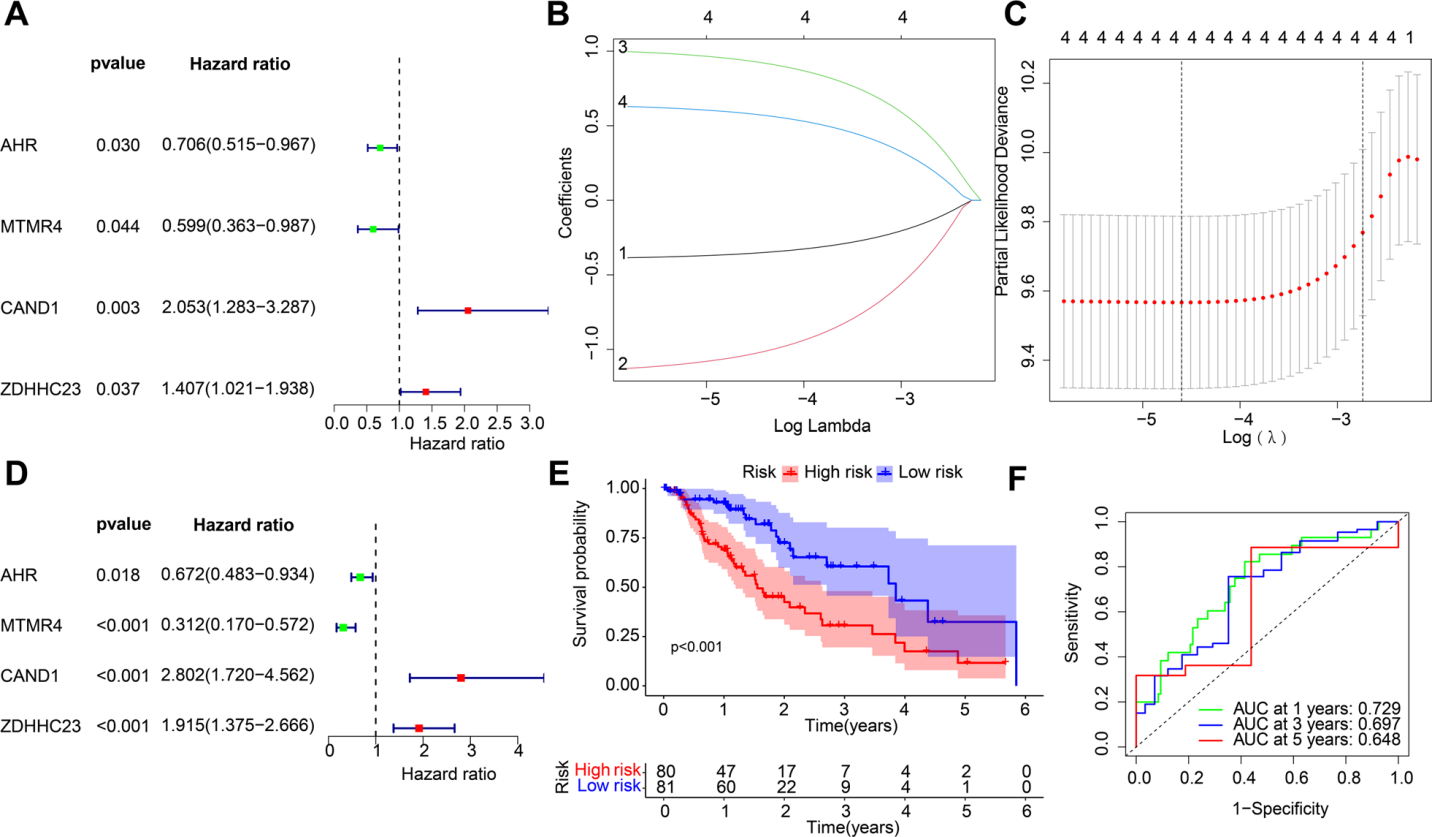
Evaluation of efficacy of the radiosensitivity-related mRNAs in the prognosis of patients with esophageal cancer. **A** Univariate Cox regression analysis for the prognosis-related mRNAs in esophageal cancer patients. The left part indicates the miRNA name, and the middle part indicates the *p* value. The Hazard ratio represents the risk rate. Risk rate greater than 1 represents a high risk of this gene, and risk rate less than 1 represents a low risk. The right part indicates the risk rate distribution. **B** LASSO coefficient distribution of 4 mRNAs s in esophageal cancer. **C** Selection of the optimal parameters (lambda) in the LASSO analysis of esophageal cancer. **D** Multivariate Cox regression analysis for the prognosis-related mRNAs in esophageal cancer patients. **E** A Kaplan-Meier survival curve analysis of patients with high- and low-risk. **F** ROC analysis of the prognostic risk model. The lower right corner indicates the AUC value

To further explore the key miRNAs and mRNAs with prognostic values in esophageal cancer patients, 2 miRNAs and 4 mRNAs were identified with association with the prognosis of patients with esophageal cancer in 21 miRNAs and 21 mRNAs in the miRNA-mRNA regulatory network by means of univariate COX analysis (Figs. 5A and 6A). Following LASSO regression analysis (Figs. 5B, C and 6B, C), a multivariate Cox analysis (Figs. 5D and 6D) was performed and highlighted two miRNAs (namely, miR-132-3p and miR-576-5p) and four mRNAs (namely, CAND1, ZDHHC23, AHR and MTMR4) could be adopted for the construction of prognostic risk models.

Patients were subsequently classified into high- and low-risk groups based on the median risk scores of miRNAs and mRNAs, and the results of Kaplan-Meier survival curve illustrated that the survival time of patients in the low-risk group was longer relative to those of patients in the high-risk group (Figs. 5E and 6E). Moreover, the results of ROC analysis also exhibited that the prognostic risk model based on two miRNAs and four mRNAs could accurately predict 1-, 2-, and 3-year overall survival (OS) in patients with esophageal cancer (Figs. 5F and 6F).

### Both miR-132-3p/CAND1/ZDHHC23 and miR-576-5p/AHR were closely associated with radiosensitivity in esophageal cancer

**Fig. 7:**
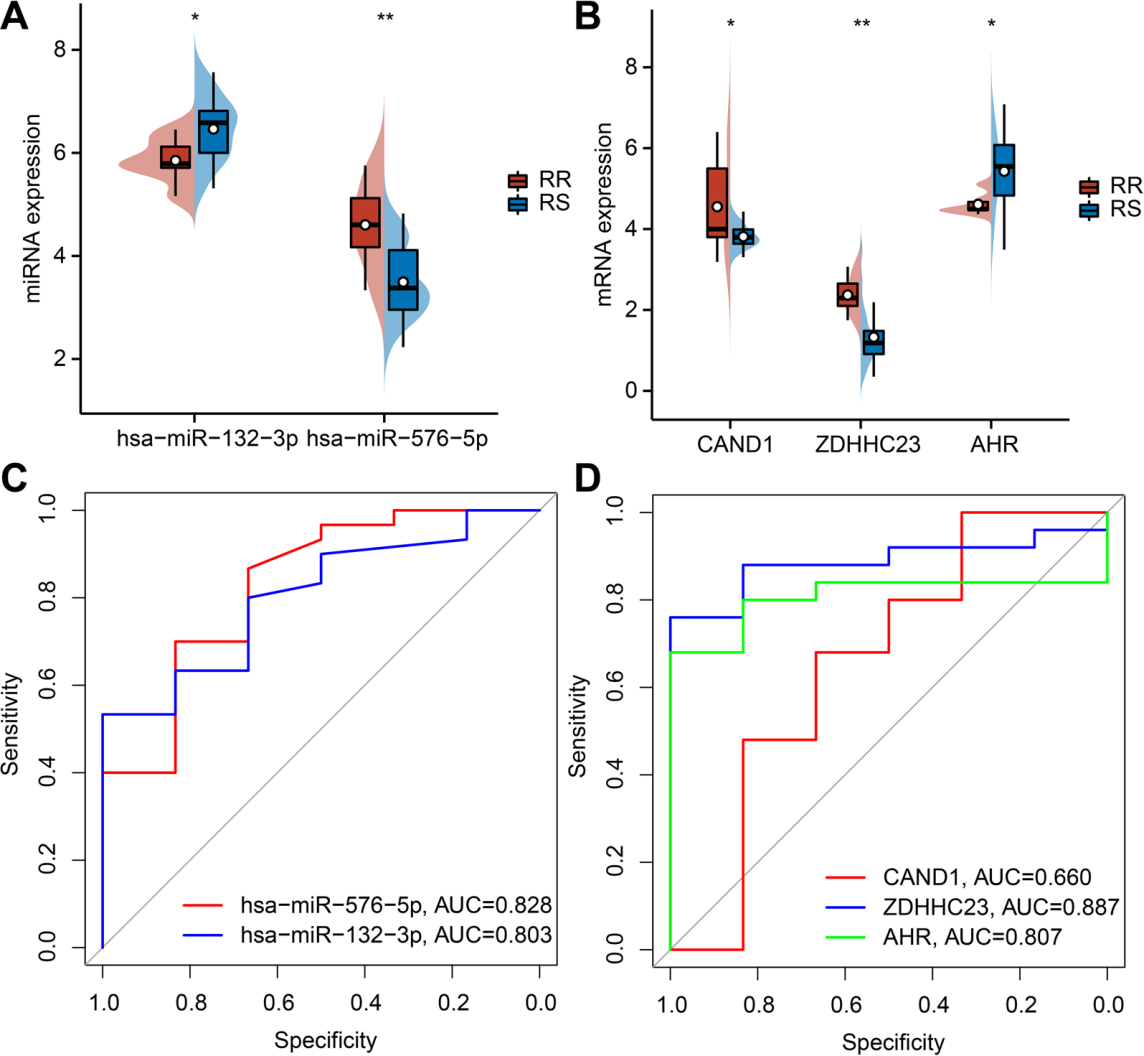
Screening and efficacy evaluation of the miRNA-mRNA regulatory network related to the radiosensitivity in esophageal cancer. **A** Distribution of DEmiRNAs between the radiosensitive and radioresistant groups; **B** Distribution of DEmRNAs between the radiosensitive and radioresistant groups. **C** ROC curve of 2 key miRNAs. **D** ROC curve of 3 key mRNAs. *p < 0.05; **p < 0.01. RR: radioresistant; RS: radiosensitive

Based on the aforementioned results, we set our efforts to explore the expression patterns of the two miRNAs and four mRNAs in the miRNA-mRNA regulatory network. Subsequent results demonstrated that miR-576-5p, CAND1 and ZDHHC23 were all up-regulated in the radioresistant group, while miR-132-3p and AHR were both up-regulated in the radiosensitive group. These results further validated that miRNAs and mRNAs were differentially expressed between the radiosensitive and radioresistant groups (Fig. 7A-B).

We further predicted the radiosensitivity in esophageal cancer using these five key factors. The results of ROC analysis identified miR-132-3p (AUC = 0.803), miR-576-5p (AUC = 0.828), CAND1 (0.660), ZDHHC23 (0.887), and AHR (AUC = 0.807) exhibited predictive potential to distinguish between the radiosensitive and radioresistant groups (Fig. 7C, D).

Altogether, the miR-132-3p/CAND1/ZDHHC23 and miR-576-5p/AHR pathways could affect radiosensitivity in esophageal cancer.

## Discussion

Esophageal cancer represents one of the most aggressive gastrointestinal malignancies in Asia, and further accompanied by an ever-increasing incidence [1, 21]. Currently, radiotherapy is regarded as the gold-standard for the treatment of esophageal cancer [3]. However, the adverse effects related to radiotherapy can seriously affect the quality of life and even result in the interruption of radiotherapy, which hampers with the curative effect and overall prognoses [22]. In lieu of the same, it is prudent to develop and adopt novel approaches to improve the radiosensitivity and the prognosis of esophageal cancer in clinical practice. Interestingly, much evidence has come to light indicating the association between biomarker investigations and gene signatures on the radiosensitivity and outcomes of patients with esophageal cancer [23–25]. Herein, the current sought to integrate multiple gene expression profile data related to esophageal cancer from the TCGA and GEO databases, and further carry out bioinformatics analyses in the selected microarrays. In the subsequent bioinformatics analysis, the results of GO analysis characterized the role of DEmRNAs in MF and BP processes, and KEGG pathway analyses highlighted 21 DEmRNAs in the miRNA-mRNA regulatory network implicated with radiosensitivity in esophageal cancer. Thereafter, we performed additional analyses for patient survival and prognoses, with the assistance of univariate Cox analysis, LASSO regression analysis, multivariate Cox analysis, and ROC analysis. Ultimately, 2 miRNAs (namely, miR-132-3p and miR-576-5p) and 4 mRNAs (namely, CAND1, ZDHHC23, AHR, and MTMR4) were obtained followed bioinformatics analyses, and possessed the potential to serve as prognostic biomarkers for radiosensitivity in esophageal cancer patients. As a gene set, the abovementioned miRNAs and mRNAs are closely-associated with radiosensitivity in esophageal cancer, and can further effectively predict the prognosis of esophageal cancer patients following radiotherapy.

Firstly, differential analyses of miRNA-seq, mRNA-seq, and mRNA datasets obtained from the TCGA and GEO databases revealed that 125 DEmiRNAs and 42 DEmRNAs were closely related to radiosensitivity in patients with esophageal cancer. Specifically, a total of 47 miRNA-mRNA pairs were identified, comprising of 21 miRNAs and 21 mRNAs, and followed by the construction of the miRNA-mRNA regulatory network. The prognostic risk model involving miRNA and mRNA has been widely adopted to predict the prognosis of patients with esophageal cancer. Additionally, previous study has indicated the use of prognostic risk factor analysis based on the interaction between miRNA and mRNA for the identification of prognostic miRNA biomarkers for esophageal cancer based on TCGA and GEO [26]. Further in accordance with our approach, prior studies have also constructed the miRNA-mRNA regulatory networks that are associated with the prognosis of patients with esophageal cancer [9]. Moreover, there is much to evidence to highlight that dysregulation of miRNAs is involved in the development and the radiosensitivity of esophageal cancer *via* regulation of their target mRNAs [12, 13, 24]. In addition, a risk assessment model based on lncRNAs, miRNAs, and mRNAs has also been constructed to screen three independent prognostic lncRNAs, thereby exploring a more effective lncRNA-miRNA-mRNA signature to predict the prognosis of esophageal cancer [27]. In our study, the results of GO and KEGG function enrichment analysis revealed that DEmRNAs in the miRNA-mRNA regulatory network may participate in the occurrence and development of esophageal cancer. The use of GO and KEGG analyses to explore the potential biological mechanisms of DEmRNAs is well-established [27]. Similarly, a prior study also reported that DEmRNAs are primarily enriched in BP, and the regulatory networks depending on DEmRNAs are constructed, which indicates potential target miRNAs and genes for future investigations of esophageal cancer [28].

Furthermore, we uncovered that the prognostic risk model based on 2 miRNAs (miR-132-3p and miR-576-5p) and 4 mRNAs (CAND1, ZDHHC23, AHR, and MTMR4) could accurately and effectively predict the prognosis of patients with esophageal cancer. Additionally, our findings indicated that the miR-132-3p/CAND1/ZDHHC23 and miR-576-5p/AHR pathways could influence radiosensitivity in esophageal cancer. Unsurprisingly, miR-132 has been previously established as a potential biomarker of prognoses in a plethora of cancers [29]. The other miRNA in focus, miR-576-5p is highly-expressed in ESCC cells, which promotes the migration and invasion of ESCC cells, underscoring that miR-576-5p could predict the prognosis and serve as a novel therapeutic target for esophageal cancer [30, 31]. On the other hand, CAND1 (Cullin-associated neural-precursor-cell-expressed developmentally down-regulated 8 (NEDD8) dissociated protein 1 (CAND1)), a regulator of cullin-RING (a fascinating novel gene) ubiquitin ligases (CRLs), is implicated in the development of cancer resistance [32, 33]. Meanwhile, NEDD8 knockdown is known to accumulate CRLs substrates through the inactivation of CRLs to inhibit the malignant phenotype, thereby serving as a therapeutic target for ESCC [34]. Furthermore, various studies have indicated the importance of AHR (aryl hydrocarbon receptor) in the occurrence and development of esophageal cancer, and further suggested AHR as a potential biomarker for esophageal cancer [35, 36]. In addition, the study performed by To *KK et al.* further explored the effects of AHR in cisplatin resistance of ESCC cells [37]. Lastly, ZDHHC23 is known to serve as a potential therapeutic target for human gliomas [38], while its role in esophageal cancer is unclear. Besides, the specific mechanisms of these miRNAs and mRNAs in regard to radiosensitivity in esophageal cancer remain to be further elucidated.

## Conclusion

**Fig. 8:**
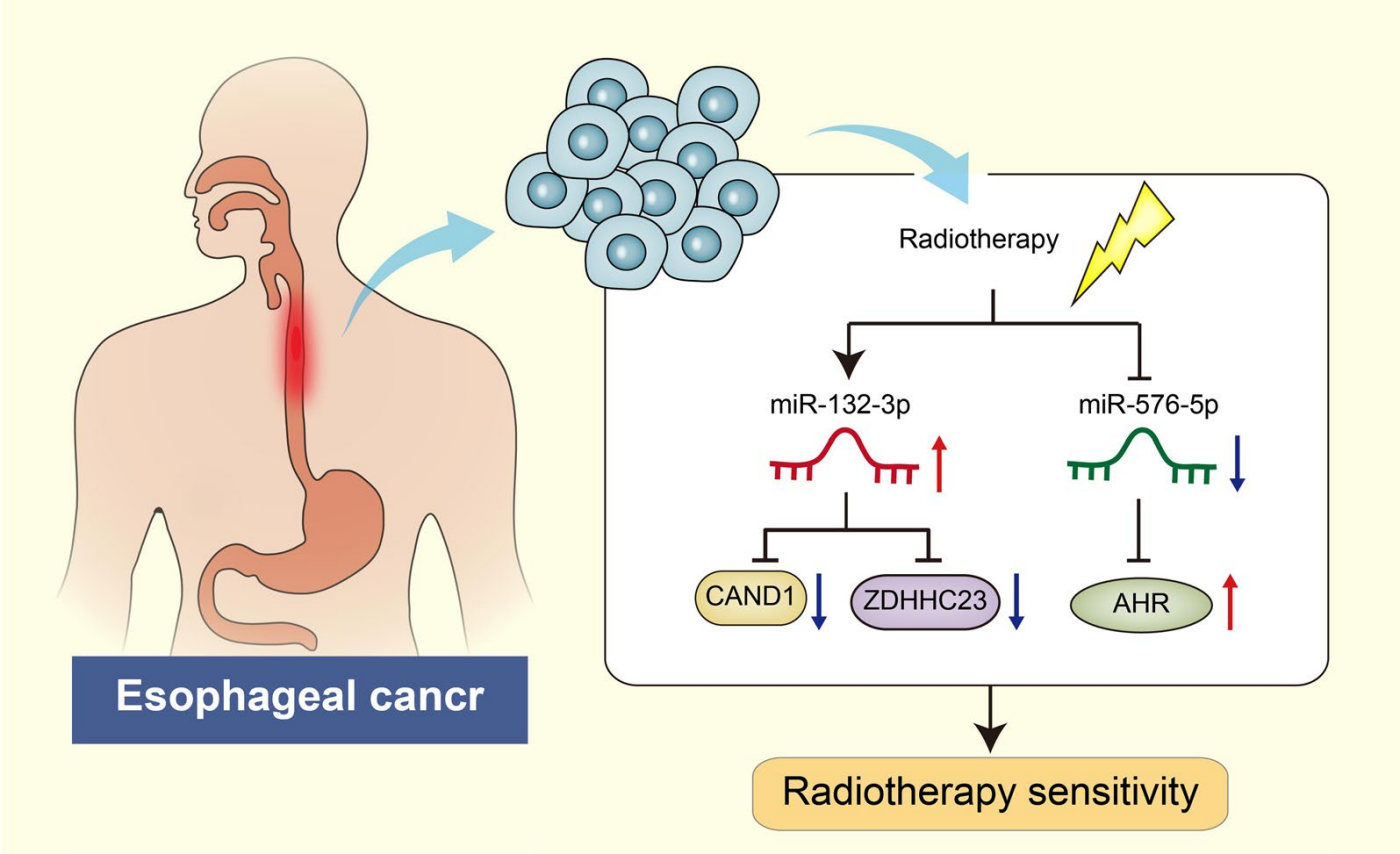
Molecular mechanism of the miRNA-mRNA regulatory network related to the radiosensitivity in esophageal cancer

Collectively, findings obtained in the current *in silico* study provide evidence for the prognostic evaluation in esophageal cancer following radiotherapy by targeting the miR-132-3p/CAND1/ZDHHC23 and miR-576-5p/AHR pathways (Fig. 8). Furthermore, a signature of these selected genes could serve as a potential prognostic indicator for the survival of patients with esophageal cancer following radiotherapy. However, our study also has some limitations. In our study, these prognostic markers were not validated in dependent cohort, so further prospective research is needed to confirm the predictive ability of these diagnostic markers, so as to scientifically provide credible theoretical basis for predicting the radiotherapy effect of esophageal cancer patients. Therefore, Future investigations are warranted with the inclusion of in vivo and in vitro experiments to validate the prognostic assessment of candidate genes in esophageal cancer after radiotherapy.

## Supplementary Information

**Additional file 1: Table 1**. GEO dataset retrieval results.

## Data Availability

The datasets generated and/or analysed during the current study are available in the Figshare, [https://figshare.com/articles/dataset/Supplemental_Result_xlsx/21397614].

## Abbreviations

ROC: Receiver operating characteristic
PD: Progressive disease
SD: Stable disease
GO: Gene Ontology
KEGG: Kyoto Encyclopedia of Genes and Genomes
OS: Overall survival
