# Supplementary material for: Identification of the miRNA-mRNA regulatory network associated with radiosensitivity in esophageal cancer based on integrative analysis of the TCGA and GEO data
Source: BMC Med Genomics. 2022 Dec 1;15:249. doi: 10.1186/s12920-022-01392-9 (PMC9714096; doi:10.1186/s12920-022-01392-9)
Supplement: Supplementary file 1 — Additional file 1: Table 1. GEO dataset retrieval results. [file 12920_2022_1392_MOESM1_ESM.docx]

**Supplementary Table 1** GEO dataset retrieval results

| Number | Series Accession | Organism | Group | |
| --- | --- | --- | --- | --- |
| 1 | GSE137867 | Homo sapiens | before radiotherapy(n=4) | after radiotherapy(n=4) |
| 2 | GSE138162 | Homo sapiens | before radiotherapy(n=1) | after radiotherapy(n=1) |
| 3 | GSE90578 | Homo sapiens | control(n=2) | stathmin overexpression(n=2) |
| 4 | GSE61816 | Homo sapiens | TE13(n=3) | TE13R(n=3) |
| 5 | GSE61772 | Homo sapiens | KY170(n=3) | KY170R(n=3) |
| 6 | GSE61620 | Homo sapiens | Seg-1(n=3) | Seg-1R(n=3) |
| 7 | GSE57083 | Homo sapiens | Esophagus cell line(n=14) | |
| 8 | GSE13898 | Homo sapiens | normal(n=28) | tumor(n=75) |
| 9 | GSE10127 | Homo sapiens | tumor(n=13) | |
